# Multi-level reproducibility of signature hubs in human interactome for breast cancer metastasis

**DOI:** 10.1186/1752-0509-4-151

**Published:** 2010-11-09

**Authors:** Chen Yao, Hongdong Li, Chenggui Zhou, Lin Zhang, Jinfeng Zou, Zheng Guo

**Affiliations:** 1 Bioinformatics Centre and Key Laboratory for NeuroInfomation of the Education Ministry of China, School of Life Science, University of Electronic Science and Technology of China, Chengdu, 610054, China; 2 Colleges of Bioinformatics Science and Technology, Harbin Medical University, Harbin 150086, China

## Abstract

**Background:**

It has been suggested that, in the human protein-protein interaction network, changes of co-expression between highly connected proteins ("hub") and their interaction neighbours might have important roles in cancer metastasis and be predictive disease signatures for patient outcome. However, for a cancer, such disease signatures identified from different studies have little overlap.

**Results:**

Here, we propose a systemic approach to evaluate the reproducibility of disease signatures at multiple levels, on the basis of some statistically testable biological models. Using two datasets for breast cancer metastasis, we showed that different signature hubs identified from different studies were highly consistent in terms of significantly sharing interaction neighbours and displaying consistent co-expression changes with their overlapping neighbours, whereas the shared interaction neighbours were significantly over-represented with known cancer genes and enriched in pathways deregulated in breast cancer pathogenesis. Then, we showed that the signature hubs identified from the two datasets were highly reproducible at the protein interaction and pathway levels in three other independent datasets.

**Conclusions:**

Our results provide a possible biological model that different signature hubs altered in different patient cohorts could disturb the same pathways associated with cancer metastasis through their interaction neighbours.

## Background

Analysis of gene expression patterns in cancers has greatly enhanced our understanding of the biology of cancer and provided a way to improve the prediction of many cancers. For example, many signature genes have been extracted from microarray data to predict the outcome of breast cancer [1-4]. However, for a particular disease, signature genes identified from different studies are usually highly inconsistent, raising doubts about the biological significance or clinical implication of the signatures identified [5-7]. In attempts to tackle this problem, many approaches have been proposed for the extraction of network-based disease signatures based on protein-protein interaction (PPI) data. Notably, because the PPI data is a union of the interactions activated under various conditions, and currently includes a lot of false positives, it alone can provide limited information for discriminating interactions in different biological pathways such as signal transduction pathways. On the other hand, considering that gene expression is sensitive to disease conditions, it is reasonable to combine gene expression data with PPI data to measure the'activity'of PPI subnetworks in response to the investigated conditions and such subnetworks are often suggestive of functional signaling cascades, metabolic pathways and molecular complexes that are associated with the disease phenotypes [8-11]. For example, Chuang *et al*. identified PPI subnetworks with coherent gene expressions as disease signatures that were suggested to be more accurate than single gene signatures for predicting breast cancer metastasis[11]. However, the subnetworks identified from different datasets were still highly inconsistent [12]. Recently, Taylor *et al*. searched for changes in the global modularity of the human interactome and found that patients who survived breast cancer had an organization of the PPI network different from that in patients who died of the illness[13]. Specifically, they suggested that "hub" proteins with altered co-expression relation with their interaction partners can be used as robust signatures to predict cancer outcome. However, as shown here, such signature hubs selected from different studies for breast cancer metastasis have little overlap.

This irreproducibility problem is usually attributed to deficiency in experimental designs, different platforms and statistical analyses of disease signatures [14,15]. However, it is very likely that the inconsistency of disease signatures discovered from different cancer samples for a particular cancer might reflect the biological variation and heterogeneity of the cancer [5,16]. It is becoming increasingly clear that, for a particular cancer, genetic and epigenetic changes in different patients are extremely heterogeneous. Especially, as demonstrated in recent high-throughput screens of somatic mutation of genes in cancer genomes, the vast majority of gene mutations are different among patients with a particular cancer [17-22]. It is also becoming clear that diverse molecular changes in cancers might actually be consistent in some essential cellular functions (hallmarks) whose alterations might collectively dictate malignant growth for almost all human cancers [23,24]. Therefore, it is reasonable to design scores to evaluate the reproducibility of disease signatures of cancers at multiple levels based on some biological assumptions (or molecular models), taking into account functional relations between the disease signatures such as expression correlation [16] and functional similarity [25]. If a score is significantly higher than expected by chance, it provides statistical evidence that the underlying model could correctly explain a large fraction of diverse but functionally related disease signatures. In this sense, the biological assumptions for designing the scores are testable.

Here, we propose a systemic approach to evaluate the reproducibility of network-based disease signatures derived for a particular cancer, taking into account their functional relations. Specifically, we evaluated the reproducibility of signature hubs for characterizing the changes of global modularity of the human interactome for breast cancer metastasis [13]. First, based on the assumption that proteins with similar interaction neighbours are likely to have similar biological functions [26,27], we proposed a topological overlap score, the percentage of overlap based on topology similarity (POT) score, to measure the reproducibility of signature hubs detected in different datasets. Using the POT score, we found signature hubs detected in two datasets for breast cancer metastasis were highly consistent in terms of frequently sharing neighbourhood proteins in the human PPI network and displaying consistent co-expression changes with the overlapping neighbours. Then, we showed that the interaction neighbour proteins shared by the two lists of signature hubs from the two datasets tended to be cancer susceptibility genes and affect some pathways known to be associated with breast cancer pathogenesis, indicating that these pathways might have important diagnostic and therapeutic implications. Finally, we proved that these results were highly reproducible in three other independent datasets for breast cancer metastasis.

## Results

### Network topology consistency of the hub protein lists

We first searched for signature hubs whose co-expressions with their interacting partners were significantly different between patients labelled non-metastatic and metastatic. We used the method proposed by Taylor *et al*. [13], as described briefly in *Methods*, in the dataset (the Wang dataset) compiled by Wang *et al*. [28] and in the dataset (the Desmedt dataset) compiled by Desmedt *et al*.[29]. Here, we did not apply the FDR control at the step of finding signature hubs because the statistical powers of most multiple test adjustment methods are decreased in the presence of wide and correlated expression changes of genes in cancers [30,31]. Instead, we used a *P *value of 0.01 to find candidate signature hubs, as in the work by Taylor *et al*.[13]. With *P *< 0.01, we identified a total of 65 and 72 signature hubs in the Wang dataset and Desmedt dataset, respectively (See Additional file 1-Table S1 for the signature hubs.). Only 4 signature hubs appeared in both datasets and the percentage of overlaps (PO) score of the hub lists was only 5.9%. Thus, at the level of individual proteins, the signature hubs detected in different studies were extremely inconsistent, although the PO score was significantly larger than expected by chance alone (hypergeometric test *P *= 0.027).

Then, we evaluated the reproducibility of two lists of signature hubs by the POT score which measures the percentage of overlapped interaction neighbours of signature hubs extracted from different studies (see *Methods*). First, by the hypergeometric distribution model, with FDR < 0.05, we tested whether the interaction neighbours of a hub in a list overlapped significantly with the neighbours of at least one of the hubs in another list. Then, considering that signature hubs with significant neighbourhood overlaps might have similar functional roles, we calculated the POT score for two lists of signature hubs. The POT score between the lists of signature hubs extracted from the Wang dataset and the Desmedt dataset was as high as 73%.

Next, we did three random experiments to test whether the increased overlap might be introduced by some factors irrelevant to the disease status. First, for each dataset, we assigned phenotype labels randomly to patients to generate expression data with the same correlation structure as the original dataset, and then searched for signature hubs in the PPI network by the approach used with the real data. Because the phenotype information was randomised, the detected signature hubs should be irrelevant to disease status. Repeating this process 1000 times, we found the average of the POT scores for the random pairs of protein lists was 41%, which was significantly smaller than the score (73%) observed with the real data (*P *< 0.005). Second, we tested whether the increased reproducibility might be due to the network topology. From the same PPI network, we randomly selected 1000 pairs of protein lists with the same lengths as the signature hub lists and then computed their POT scores. The average of the POT scores for these random pairs of protein lists was 44%, which was significantly smaller than that observed (*P *< 0.005). Third, we tested whether the high level of reproducibility might be due to the high degrees (numbers of interaction partners) of signature hubs. Using a local rewiring algorithm [32], we produced 1000 random PPI networks in each of which all proteins had exactly the same connectivity as in the original PPI network and the choice of their interaction partners was random. Then, from each random network we selected the pairs of hub lists that had exactly the same lengths and degree distributions as the two lists of signature hubs extracted from the actual PPI network. Then, we recalculated the POT score for this random pair of hub lists. This process was repeated 1000 times. The average POT score for 1000 pairs of random hub lists was 42%, significantly smaller than that observed (*P *< 0.005).

Both false negatives and false positives are concerned for the PPI data quality [33,34]. To tackle the low coverage problem introduced by false negatives, we integrated 8 databases to generate a large PPI network for our study. To reduce the effect of false positives, we also used a small PPI network which contained only the hand-curated PPI interaction data from OPHID [35] and MINT [36]. The POT score was decreased a little to 62% due to the smaller network size based on this PPI dataset. However, the POT score was significantly higher than those (20%, 29% and 17%) based on each of the three random experiments described above (*P *< 0.005), respectively. Two PPI networks generated similar POT scores, suggesting that our results were rather robust against false negatives and false positives in the PPI data.

### Pathway consistency of the hub protein lists

If two signature hubs share many interaction neighbour proteins, then they might participate in the same or similar functions [26,27]. To reveal the consistency of signature hub lists at the pathway level, for each signature hub identified from each dataset, we analysed the enrichment of its interaction neighbours in pathways collected in the Kyoto Encyclopaedia of Genes and Genomes (KEGG) [37] (see *Methods*). With FDR < 0.01, we found that 34 pathways were enriched significantly with the neighbours of at least one of the signature hubs detected in the Desmedt dataset, among which 26 pathways were included in the 38 significant pathways detected in the Wang dataset (See Additional file 1-Table S2 for the list of 26 pathways.). Notably, among the other 12 pathways detected in the Wang dataset but not in the Desmedt dataset, 11 were marginally significant in the Desmedt dataset with *P *< 0.05. Similarly, among the 8 pathways detected in the Desmedt dataset but not in the Wang dataset, 6 were marginally significant in the Wang dataset with *P *< 0.05. Thus, some inconsistency between the two datasets might come from a reduction of the statistical power by using the stringent FDR control for adjusting multiple tests when the multiple tests are not independent of each other [30,31].

We did a random experiment to test the significance of the high concordance of pathway enrichment (see *Methods*). First, we took the 38 pathways identified from the Wang dataset as the gold standard. From each of the random networks produced by a local rewiring algorithm [32], we extracted a random hub list of the same length and degree distribution with the list of signature hubs identified from the Desmedt dataset. Then, we detected the pathways enriched with the neighbours of random hubs and compared them with the gold standard. Repeating this process 1000 times, we found the average number of overlapping pathways was 1, significantly fewer than the 26 overlaps observed in the real data (*P *< 0.001). The result was the same when taking the pathways detected from the Desmedt dataset as the gold standard.

The 26 pathways detected in both datasets included many pathways known to be deregulated in breast cancer pathogenesis, such as cell cycle, apoptosis, Jak-STAT, MAPK, ErbB, Wnt and P53 signalling pathways [38]. Among these 26 pathways, there were 191 and 238 interaction neighbours of the signature hubs identified from the Wang and Desmedt datasets, respectively, and they shared 114 proteins, which was significantly more than expected by chance alone (hypergeometric test *P *< 2.2 × 10^-16^). These common interaction neighbour proteins might have important roles in cancer. To test this, we assembled a list of 427 cancer susceptibility genes from the Cancer Gene Census database [39] and found 50 out of 114 neighbour proteins were known cancer proteins (hypergeometric test *P*= 6 × 10^-4^). When using the 685 genes collected in our F-census database [25], 100 out of 114 neighbour proteins were included (hypergeometric test with *P *< 2.2 × 10^-16^).

**Figure 1:**
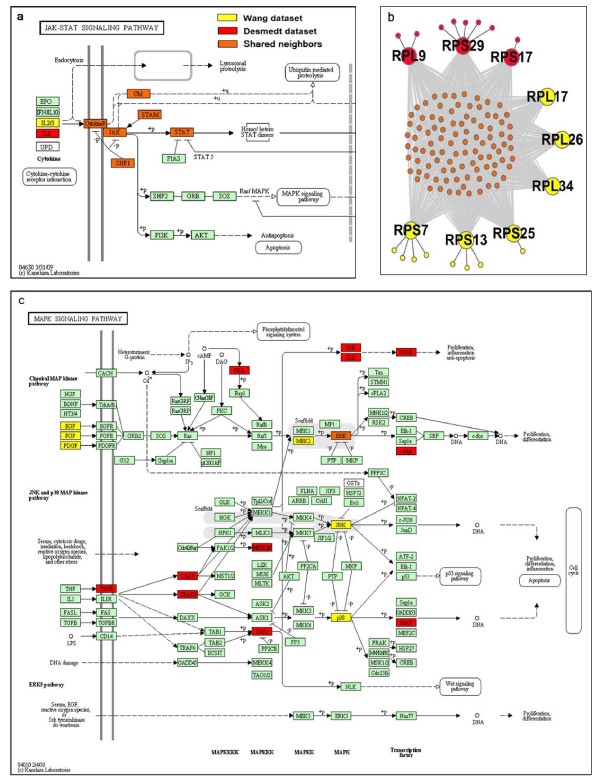
**Examples of pathways shared by a signature hub from the Wang dataset and a signature hub from the Desmedt dataset**. (a) JAK-STAT signaling pathway; (b) Ribosome complex; (c) MAPK signaling pathway. The yellow and red colors represent proteins (both hubs and their neighbors) identified from the Wang and Desmedt datasets, respectively. The orange colors represent the overlapped neighbors of these two hub proteins. Please see the main text for detailed explanation.

The above results suggested that the two lists of signature hubs might affect the same pathways. In one situation, in different cohort patients, a cancer-associated pathway could be affected by the co-expression changes of different signature hubs with the same set of neighbours enriched in this pathway. For example (Figure 1a), the interleukins IL2 and IL6 were identified as signature hubs from the Wang and Desmedt datasets separately and their overlapped neighbours were enriched in the Jak-STAT signalling pathway. Thus, changes of co-expression of these shared neighbours with either IL2 or IL6 might disrupt the Jak-STAT signalling pathway and contribute to the progression of cancer [40]. For another example (Figure 1b), 6 signature hubs identified from the Wang dataset and another 3 signature hubs identified from the Dsemedt dataset are all subunits of a ribosome complex for protein biosynthesis. They share other subunits as interaction neighbours and their deregulation might be associated with cell growth and proliferation [41]. In another situation, a cancer-associated pathway could be affected by changes of different signature hubs interacting with different sets of neighbours that were separately enriched in this pathway. For example (Figure 1c), proteins DUSP3 with degree 18 and CAD with degree 39 were identified as signature hubs in the Wang and Desmedt datasets separately. The neighbours of each of these two proteins were enriched in the MAPK signalling pathway associated with cancer metastasis [42], but their neighbours shared only 1 protein. It has been suggested that DUSP3 can negatively regulate members of the MAP kinase superfamily (MAPK) [43], while the deregulation of CAD proteins might be associated with activation of the MAPK cascade[44]. Notably, this functional relation between two signature hubs was not reflected by the POT score, which considers only overlapping neighbours between the signature hubs (see *Discussion*).

### Co-expression consistency of the hub proteins lists

Considering that a signature hub disturbs functions through differential co-expression with their interaction neighbours [13], we further assumed that two functionally similar hubs should display consistent co-expression changes with their overlapping neighbours across different datasets [45,46]. Therefore, for two hubs detected from two datasets separately, we additionally tested the consistency of the directions of their correlations with the shared neighbours across the datasets by the Bernoulli distribution model (see *Methods*).

With the co-expression restriction, for Wang and Desmedt dataset, the POT score (denoted as POG-e score) decreased a little from 73% to 67%, largely explainable when considering that any extra restriction may miss some true relations. On the other hand, the random POT-e score decreased greatly from 44% to 26%. The results suggested that signature hubs sharing neighbours were significantly consistent in the change directions of correlations with their shared neighbors. For example, from the Wang and Desmedt datasets separately, the interleukins IL2 and IL6 were identified as signature hubs and their 6 overlapped neighbours were enriched in the Jak-STAT signaling pathway. In both Wang and Desmedt datasets, the expressions of IL2 and IL6 were both positively correlated with the expressions of these shared neighbours in non-metastatic patients, but negatively correlated with the expressions of the shared neighbours in metastatic patients. These results suggest that Jak-STAT signaling pathway could be perturbed by the disruption of co-expressions of either IL2 or IL6 with the shared neighbours during the breast cancer metastasis.

### Validation in three independent breast cancer datasets

We validated our results by analyzing three other independent datasets for breast cancer metastasis [2,47,48]. For lists of signature hubs extracted from every two breast cancer datasets, the PO score was less than 4%. However, the corresponding POT scores took values ranging from 61% to 75% which were all significantly larger than expected by chance according to the three random experiments as described in *Methods*. Similar results were observed based on the POT-e score (*P*< 0.005, see Additional file 1- Table S3 for details).

For example, 80 signature hubs were identified from the Vijver dataset, among which only 4 and 1 overlapped with the signature hubs found in the Wang and Desmedt datasets, respectively. However, the corresponding POT scores were 64% and 75%, respectively, and they were both significantly larger than expected by chance (*P *< 0.005), according to each of the three random experiments as described in *Methods*. Notably, although the average POT score between the Wang and Vijver datasets was only 64%, the POT score for the signature hub list extracted from the Vijver dataset to the signature hub list extracted from the Wang dataset was 71%, suggesting that many of the signature hubs detected from the Vijver dataset could be represented by the signature hubs from the Wang dataset in terms of neighbourhood similarity. The score in the opposite direction was only 57%, indicating that the samples used in the Vijver dataset might be insufficient for capturing enough signature hubs to cover the signature hubs extracted from the Wang dataset.

According to pathway enrichment analysis, the signature hubs extracted from the Vijver dataset and those from both the Wang dataset and the Desmedt dataset were highly consistent. Among the 26 pathways shared by the Wang and Desmedt datasets, 19 were included in the 34 pathways identified from the Vijver dataset, significantly more than expected by chance alone (hypergeometric test *P*= 5.2 × 10^-5^). All the other 7 pathways detected in both the Wang and Desmedt datasets were marginally significant in the Vijver dataset with *P *< 0.05. These results indicated that these pathways, such as MAPK signaling and apoptosis pathways which were also founded in other studies [11,49], might be disturbed in the breast metastatic progression.

The above results confirmed that signature hubs detected from different datasets for breast cancer metastasis were reproducible in terms of neighbourhood protein overlap and, more generally, pathway overlap. Notably, approximately half of the patients in the Vijver dataset were lymph node-positive and underwent adjuvant therapy before expression profiling, whereas all patients in the Wang dataset had lymph node-negative breast cancer [11]. However, our results indicated that the two types of samples might have similar molecular changes at the pathway level.

## Discussion

Changes in the global modularity of the human interactome might provide important insights into the mechanism underlying cancer metastasis [13]. As shown in this study, although signature hubs detected from different studies for breast cancer metastasis have little overlap, they are highly consistent in terms of frequently sharing interaction proteins and displaying consistent co-expression changes with their overlapping neighbours, indicating that they might alter the same pathways through differential co-expression with their interaction neighbours. To some extent, this finding is similar to the observation made in microRNA studies that a cancer pathway could be changed in cancer cases by either aberration of some cancer genes or modification of microRNAs regulating these genes[50]. Recently, using several microarray datasets, Li *et al*. identified gene signature modules with high predictive accuracy for breast cancer metastasis[49]. These modules contained two parts: a set of signature genes that are dynamically modulated between 'high-risk' and 'low-risk' patients and a unique set of cancer driver-mutating genes that are the direct protein interacting partners of the signature genes. At the conceptual level, their results also suggested that, despite low overlap, disease signatures detected from different datasets may reflect consistent function disruptions. Especially, many modules identified by Li *et al*., such as cell cycle, apoptosis and immune response, were functionally consistent with our KEGG pathways enriched with proteins targeted by different signature hubs.

The POT score proposed in this paper considers the functional concordance between signature hubs only according to their overlapping neighbours. The significantly high POT scores between signature hubs derived from different studies for breast cancer metastasis indicates that the biological assumption included in this score could explain a large fraction of diverse signature hubs. However, the POT scores were only about 70% for the five datasets in this study and some inconsistent signature hubs could not be explained by this model. One explanation is that the incomplete PPI data might be insufficient for capturing all functional links among signature hubs. Another possibility is that there might be other molecular models that can explain the remaining inconsistent discoveries. For example, as illustrated by a case presented in *Results*, two signature hubs with non-overlapping neighbours might be functionally consistent if their neighbours are enriched separately in the same pathway, but such a functional relation is not measured by the POT score. Principally, we could further consider this and other possible relations of signature hubs to reveal the consistency of signature hub lists. For example, we could evaluate the consistency of signature hub lists at the pathway level by counting overlaps of the enrichment pathways associated with different signature hub lists. However, such a pathway level analysis might have only a limited application scope because many proteins have not been annotated to current pathway databases such as KEGG used in this study. The limited annotation can reduce the power of finding true enrichment pathways and introduce some inconsistency [11]. More problematically, pathways defined in current databases are often inconsistent and their boundaries are unclear [51]. For example, it is possible that a pathway documented in a pathway database consists of several sub-pathways, and only alterations of genes within a sub-pathway have the same or a similar role in cancer development, and the genes within the other sub-pathways might be irrelevant to, or have other roles, in the disease mechanism. In such a situation, it would be ambiguous if we consider two signature hubs as functionally equivalent (reproducible) when they are associated with different parts of the pathway through their interaction proteins. Thus, to interpret the consistency of signature hubs at the pathway level, we need to determine pathways or their sub-pathways that are most relevant to a disease. Compared with KEGG and other pathway databases, Gene Ontology (GO)[52] could help us tackle this problem to some degree because it describes biological functions from general to specific in a hierarchy. However, currently, it is still a difficult task to treat the redundant annotations in GO properly [53,54] and this problem deserves future research efforts [51,55]. Thus, currently, the pathway analysis can only partially support the POT score analysis. When the pathway definition and gene annotation are improved, the pathway analysis will become an efficient way of explaining inconsistent signatures generated from different studies.

The irreproducibility of molecular signatures detected for a complex disease is also a common problem in many other research areas based on high-throughput biotechnology such as proteomics [56] and metabolomics [57]. Also, it is very likely that the small samples typically used in current studies of these areas might reflect the wide and diverse molecular changes in a complex disease only partially. In general, taking into account the diverse but correlated molecular changes in a complex disease such as a cancer, our approach provides a framework for explaining the reproducibility of biological findings at the systems biology level. However, even when we could find functionally consistent disease signatures from currently available samples, it might still need thousands of samples to find a few reproducible individual signatures. Thus, it would be a difficult task to build a consensus prognostic classifier on the basis of a few signatures for a complex disease [6]. To circumvent the difficulty of finding consistent signatures themselves, we could use some biological pathways commonly affected by diverse molecular changes as modular signatures to build robust diagnostic classifiers [58]. The identification of such clearly defined key pathways of cancer metastasis might provide crucial guidance for designing diagnostic classifiers and, perhaps, appropriate drug combinations [59].

## Conclusions

Distant metastases are the major cause of death in cancer patients. The heterogeneous nature of tumours leads to different responses from different patients with the same type of cancer. Therefore, as a sign that two studies have detected the same result for a disease, it is not necessary that the signature lists themselves are consistent. They could be probably tracking a common set of biologic phenotype, as we shown here, in protein network, signature hubs with low reproducibility may actually have similar functions by interacting with the same sets of neighbour proteins.

## Methods

### Datasets

Five datasets of gene expression profiles for breast cancer metastasis are described in Table 1. Patients who had been detected metastasis within 5 years during follow-up visits were assigned to metastatic and the remaining patients were assigned to non-metastatic. We mainly analyzed the Wang dataset [28] and Desmedt dataset [29], and the other three datasets [2,47,48] were used for validation.

**Table 1:** The five datasets analyzed in this study

Datasets ^a^	No. of Patients	metastatic	non-metastatic	Platforms
Wang dataset[28]	286	106	180	Affymetrix HG-U133a

Desmedt dataset[29]	198	35	163	Affymetrix HG-U133a

GSE1456[47]	159	40	119	Affymetrix HG-U133a

GSE3494[48]	218	37	181	Affymetrix HG-U133a

Vijve dataset[2]	295	78	217	Agilent Hu25K

^a ^Datasets were named according to the authors or GEO numbers.

The human PPI data were downloaded from MINT [36], BIND [34], IntAct [60], HPRD [61], MIPS [62], DIP [63], KEGG (PPrel for protein-protein interactions, ECrel for enzymes involved in neighboring steps) [64] and Reactome [65]. To increase the coverage of the PPI network, we pooled together these 8 PPI datasets to construct an integrated PPI network that consists of 101,729 distinct interactions involving 12,372 human proteins [66]. We restricted our analysis to the 5470 genes encoding proteins in this PPI network and presenting in all five breast datasets. We also did the analysis using the OPHID data [35] combined with MINT data [36], which was the same as the PPI data used by Taylor *et al*.[13].

The 60 pathways analysed here were collected from the categories "Environmental Information Processing" and "Cellular Processes" in the KEGG database [37] at March 2010.

### Selection of signature hubs

As Taylor *et al*. did, proteins with at least 3 interaction neighbours in the PPI network were defined as hubs and used for further study [13]. To determine the difference of co-expression of a hub with its interaction partners between metastatic and non-metastatic patients in a dataset, we calculated the Pearson correlation coefficients (PCCs) between this hub and its interaction partners in each patient group and then the absolute difference of the PCCs between two groups. We randomly permuted patients in the two groups 1000 times to calculate the random distribution of the absolute difference of PCCs between the groups. Then the real absolute difference of the PCCs for this hub between patient groups was compared to the random distribution to generate its *P *value. Hub proteins with *P *values < 0.01 were selected as candidate signature hubs, also referred to as signature hubs for short in the text.

### Multi-level evaluation of reproducibility

In the following, we describe some scores for measuring the consistency between signature hubs derived from different studies for breast cancer metastasis.

At the individual protein level, the consistency of two lists of signature hubs was measured by the percentage of overlaps (PO) score [30]. Suppose hub list 1 with length *L*_1 _and list 2 with length *L*_2 _share *k *proteins, then the POG score from list 1 (or 2) to list 2 (or 1) is:

$${\text{Po}}_{12}\text{=}k/L_{1}$$

$${\text{Po}}_{21}\text{=}k/L_{2}$$

The average of the scores in the two directions is:

$$\text{PO}\text{=}\left(PO_{12}\;+\;PO_{21}\right)/2$$

Based on the assumption that proteins with significantly overlapped interaction neighbours are likely to share the same function [26,27], we designed a score, named the percentage of overlap based on topology (POT) similarity score, to measure the consistency of two lists of signature hubs at the PPI topology similarity level. Let *n *and *m *be the number of neighbours interacting with proteins *i *and *j*, respectively, and *g *is the number of neighbours shared by these two proteins, the probability *P *of observing no fewer than *g *neighbours shared by proteins *i *and *j *by chance was calculated by the hypergeometric distribution model as[67]:

$$P=1-\sum_{i=0}^{g-1}\frac{C_{n}^{i}C_{N-n}^{m-i}}{C_{N}^{m}}$$

where *N *is the number of proteins with both PPI and gene expression data. With FDR < 0.05, the *P *value was adjusted by the Bonferroni-Hochberg procedure to account for multiple tests [68].

Then, let *T*_12 _(or *T*_21_) be the number of proteins in list 1 (or list 2) whose neighbours are overlapped significantly with the neighbours of at least one of the proteins in list 2 (or list 1), then the POT score is defined as the average of the scores in the two directions:

$$\text{POT}\;=\;\;\left({\text{POT}}_{\text{12}}\;+\;{\text{POT}}_{\text{21}}\right)/2$$

The score in one direction is:

$${\text{POT}}_{\text{12}}\;=\;\left(k+T_{\text{12}}\right)/L_{1}$$

and the score in the other direction is:

$${\text{POT}}_{21}\;=\;\left(k+T_{21}\right)/L_{2}$$

The significance of a PO score was calculated as the probability of observing at least *k *overlapped proteins by chance using the hypergeometric probability model [30]. The significance of an observed POT score was assessed by three random experiments, testing whether the POT score might be due to (1) the correlation structures of expression profiles, (2) the PPI network topology or (3) the degree distribution of the signature hubs. The details of the experimental procedures are described in *Results*.

Both the PO and POT scores are dependent on the list lengths. In this study, our major objective was to find consistent results from the hub lists detected from different studies for a disease and we did not intend to compare the consistency level of hub lists with different lengths. Thus, we did not normalize PO or POT scores as we did in our earlier work [16].

Considering the basic assumption that signature hubs may disturb functions through differential co-expression with their interaction neighbours[13], we further assumed that two functionally similar hubs should display consistent co-expression changes with their overlapping neighbours across different datasets [45,46]. Let *n *be the number of neighbours shared by two signature hubs detected separately from two datasets, and *k *is the number of these shared neighbours whose correlation changes with these two hubs are in the same direction across the two datasets. Then, the probability *P *of observing no fewer than *k *neighbours with the same directions of correlation changes by chance was calculated by the Bernoulli distribution model as:

$$P=1-\sum_{i=0}^{k-1}\left(\begin{array}{c} n\\ k-1 \end{array}\right)p^{k-1}{\left(1-p\right)}^{n-k+1}$$

With *P *< 0.05, we calculated the POT score with co-expression restriction, denoted as POT-e. Then, we did a random experiment to determine if an observed POT-e score is significantly larger than expected by chance when the change directions of correlations between hubs and their shared neighbours are irrelevant to the disease conditions. We randomly reassigned phenotype labels of samples to generate expression data with the same correlation structure as the original data, and then recalculated the POT-e score. This process was repeated 1000 times and the *P *value was calculated as, among the scores of the 1000 datasets with random phenotypes, the proportion of the scores exceeding the observed one.

### Pathway enrichment analysis

For each signature hub, we detected pathways enriched with its interaction neighbours by the hypergeometric probability model [67]. The *P *value was adjusted by the Bonferroni-Hochberg procedure with FDR < 0.01 [68].

For a disease, we took the pathways enriched with the neighbours of signature hubs detected from one dataset as the gold standard, and then calculated the overlap with the pathways enriched with the neighbours of signature hubs detected from another dataset. To test the significance of the observed overlapping, we produced a random network by using a local rewiring algorithm [32]. In the random PPI network, all proteins had exactly the same connectivity as in the original PPI network and the choice of their interaction partners was random. Then, from the random network we selected a random protein list with the same length and degree distribution as the list of signature hubs identified from another dataset. Then, with FDR < 0.01, we detected the pathways enriched with random hubs and compared them with the gold standard. Repeating this process 1000 times, we calculated the *P *values for the observed overlaps.

### Authors' contributions

CY and ZG made contributions to the concepts, CY, HDL and CGZ carried out the analyses of the data. LZ and JFZ helped to interpret the results. CY and ZG drafted the paper, and all authors read and approved the final manuscript.

## Supplementary Material

Additional file 1 **Supplemental Tables**. This file contains Tables S1-S3. Table S1 Two hub protein lists separately identified from Wang and Desmedt datasets. Table S2 List of 26 common KEGG pathways. Table S3 POT and POT-e scores for five breast cancer datasets Click here for file
