# Supplementary material for: Multi-level reproducibility of signature hubs in human interactome for breast cancer metastasis
Source: BMC Syst Biol. 2010 Nov 9;4:151. doi: 10.1186/1752-0509-4-151 (PMC2990745; doi:10.1186/1752-0509-4-151)
Supplement: Additional file 1 — Supplemental Tables. This file contains Tables S1-S3. Table S1 Two hub protein lists separately identified from Wang and Desmedt datasets. Table S2 List of 26 common KEGG pathways. Table S3 POT and POT-e scores for five breast cancer datasets [file 1752-0509-4-151-S1.DOC]

**Table S1 Two hub protein lists separately identified from Wang and Desmedt** datasets

| **Wang dataset** | | | **Desmedt dataset** | | |
| --- | --- | --- | --- | --- | --- |
| Entrez | Symbol | Degree | Entrez | Symbol | Degree |
| 53 | ACP2 | 5 | 131 | ADH7 | 13 |
| 89 | ACTN3 | 12 | 332 | BIRC5 | 17 |
| 332 | BIRC5 | 17 | 472 | ATM | 43 |
| 443 | ASPA | 9 | 688 | KLF5 | 14 |
| 641 | BLM | 17 | 701 | BUB1B | 28 |
| 819 | CAMLG | 3 | 790 | CAD | 39 |
| 890 | CCNA2 | 31 | 833 | CARS | 6 |
| 983 | CDC2 | 123 | 836 | CASP3 | 111 |
| 990 | CDC6 | 41 | 891 | CCNB1 | 58 |
| 1018 | CDK3 | 11 | 983 | CDC2 | 123 |
| 1182 | CLCN3 | 7 | 990 | CDC6 | 41 |
| 1355 | COX15 | 3 | 991 | CDC20 | 31 |
| 1837 | DTNA | 10 | 993 | CDC25A | 51 |
| 1845 | DUSP3 | 18 | 995 | CDC25C | 34 |
| 1871 | E2F3 | 30 | 1005 | CDH7 | 5 |
| 1984 | EIF5A | 4 | 1030 | CDKN2B | 6 |
| 2010 | EMD | 9 | 1124 | CHN2 | 32 |
| 2052 | EPHX1 | 24 | 1642 | DDB1 | 38 |
| 2891 | GRIA2 | 19 | 1974 | EIF4A2 | 101 |
| 2914 | GRM4 | 4 | 2100 | ESR2 | 25 |
| 2932 | GSK3B | 86 | 2592 | GALT | 10 |
| 3014 | H2AFX | 12 | 2661 | GDF9 | 22 |
| 3206 | HOXA10 | 7 | 2745 | GLRX | 9 |
| 3559 | IL2RA | 15 | 2925 | GRPR | 5 |
| 3609 | ILF3 | 10 | 2957 | GTF2A1 | 30 |
| 3619 | INCENP | 7 | 2965 | GTF2H1 | 85 |
| 3693 | ITGB5 | 28 | 2997 | GYS1 | 18 |
| 3778 | KCNMA1 | 5 | 3336 | HSPE1 | 20 |
| 3858 | KRT10 | 8 | 3570 | IL6R | 13 |
| 3866 | KRT15 | 16 | 3964 | LGALS8 | 5 |
| 3934 | LCN2 | 5 | 3981 | LIG4 | 5 |
| 4225 | MEP1B | 8 | 4000 | LMNA | 25 |
| 4288 | MKI67 | 7 | 4001 | LMNB1 | 14 |
| 4772 | NFATC1 | 14 | 4137 | MAPT | 41 |
| 4856 | NOV | 8 | 4171 | MCM2 | 43 |
| 4908 | NTF3 | 4 | 4174 | MCM5 | 45 |
| 5281 | PIGF | 5 | 4175 | MCM6 | 36 |
| 5395 | PMS2 | 5 | 4176 | MCM7 | 49 |
| 5451 | POU2F1 | 41 | 4237 | MFAP2 | 5 |
| 5524 | PPP2R4 | 6 | 4722 | NDUFS3 | 61 |
| **Wang dataset** | | | **Desmedt dataset** | | |
| Entrez Symbol Degree | | | Entrez Symbol Degree | | |
| 5757 | PTMA | 22 | 5053 | PAH | 5 |
| 5915 | RARB | 11 | 5338 | PLD2 | 52 |
| 5916 | RARG | 9 | 5609 | MAP2K7 | 28 |
| 6139 | RPL17 | 92 | 5662 | PSD | 5 |
| 6154 | RPL26 | 95 | 5706 | PSMC6 | 42 |
| 6164 | RPL34 | 93 | 5916 | RARG | 9 |
| 6201 | RPS7 | 101 | 5932 | RBBP8 | 15 |
| 6207 | RPS13 | 104 | 6005 | RHAG | 4 |
| 6230 | RPS25 | 101 | 6133 | RPL9 | 96 |
| 6388 | SDF2 | 3 | 6218 | RPS17 | 100 |
| 6427 | SFRS2 | 70 | 6235 | RPS29 | 103 |
| 6455 | SH3GL1 | 7 | 6240 | RRM1 | 26 |
| 6494 | SIPA1 | 3 | 6241 | RRM2 | 21 |
| 7112 | TMPO | 9 | 6284 | S100A13 | 3 |
| 7257 | TSNAX | 6 | 6285 | S100B | 16 |
| 8048 | CSRP3 | 6 | 6329 | SCN4A | 8 |
| 8491 | MAP4K3 | 3 | 6569 | SLC34A1 | 4 |
| 9616 | RNF7 | 10 | 6843 | VAMP1 | 8 |
| 9794 | MAML1 | 4 | 7110 | TMF1 | 8 |
| 10516 | FBLN5 | 5 | 7253 | TSHR | 21 |
| 10725 | NFAT5 | 10 | 7515 | XRCC1 | 8 |
| 11144 | DMC1 | 5 | 7639 | ZNF85 | 4 |
| 22795 | NID2 | 5 | 8120 | AP3B2 | 4 |
| 23229 | ARHGEF9 | 23 | 8318 | CDC45L | 31 |
| 30835 | CD209 | 3 | 9133 | CCNB2 | 16 |
|  |  |  | 10912 | GADD45G | 24 |
|  |  |  | 10971 | YWHAQ | 67 |
|  |  |  | 23594 | ORC6L | 32 |
|  |  |  | 23624 | CBLC | 46 |
|  |  |  | 27113 | BBC3 | 9 |
|  |  |  | 29767 | TMOD2 | 5 |
|  |  |  | 80781 | COL18A1 | 6 |

**Table S2 List of 26 common KEGG pathways**

| **Pathways** | **KEGG–ID** |
| --- | --- |
| PPAR signaling pathway | hsa03320 |
| MAPK signaling pathway | hsa04010 |
| ErbB signaling pathway | hsa04012 |
| Chemokine signaling pathway | hsa04062 |
| Cell cycle | hsa04110 |
| Oocyte meiosis | hsa04114 |
| p53 signaling pathway | hsa04115 |
| mTOR signaling pathway | hsa04150 |
| Apoptosis | hsa04210 |
| Wnt signaling pathway | hsa04310 |
| Axon guidance | hsa04360 |
| VEGF signaling pathway | hsa04370 |
| Focal adhesion | hsa04510 |
| NOD-like receptor signaling pathway | hsa04621 |
| RIG-I-like receptor signaling pathway | hsa04622 |
| Jak-STAT signaling pathway | hsa04630 |
| Natural killer cell mediated cytotoxicity | hsa04650 |
| T cell receptor signaling pathway | hsa04660 |
| B cell receptor signaling pathway | hsa04662 |
| Fc epsilon RI signaling pathway | hsa04664 |
| Neurotrophin signaling pathway | hsa04722 |
| Regulation of actin cytoskeleton | hsa04810 |
| Insulin signaling pathway | hsa04910 |
| GnRH signaling pathway | hsa04912 |
| Progesterone-mediated oocyte maturation | hsa04914 |
| Adipocytokine signaling pathway | hsa04920 |

**Table S3** POT and POT-e scores for five breast cancer datasets

| **Datasets** | **No. of Overlapping signature hubs** | **POT**  **(Random POT** ^b^**)** | **POT-e**  **(Random POT-e** ^c^**)** |
| --- | --- | --- | --- |
| Wang--Desmedt | 4 | 73% (44%) | 67% (26%) |
| Wang--Vijver | 4 | 64% (36%) | 62% (18%) |
| Desmedt--Vijver | 1 | 75% (45%) | 70% (21%) |
| Wang--GSE1456 | 1 | 67% (37%) | 61% (20%) |
| Desmedt--GSE1456 | 0 | 64% (37%) | 56% (18%) |
| Wang--GSE3494 | 2 | 61% (32%) | 53% (17%) |
| Dsemedt--GSE3494 | 1 | 73% (42%) | 68% (25%) |

^b^ Random POT scores are generated by randomly selecting 1000 pairs of protein lists with the same lengths as the signature hub lists.

^c^ POT-e and Random POT-e scores are generated by integrating PPI and gene expression data.
